# Development of a 3D printed surgical guide for Brugada syndrome substrate ablation

**DOI:** 10.3389/fcvm.2022.1029685

**Published:** 2022-11-15

**Authors:** Giacomo Talevi, Luigi Pannone, Cinzia Monaco, Edoardo Bori, Ida Anna Cappello, Mara Candelari, Robbert Ramak, Mark La Meir, Ali Gharaviri, Gian Battista Chierchia, Bernardo Innocenti, Carlo de Asmundis

**Affiliations:** ^1^Heart Rhythm Management Centre, Postgraduate Program in Cardiac Electrophysiology and Pacing, Universitair Ziekenhuis Brussel - Vrije Universiteit Brussel, European Reference Networks Guard-Heart, Brussels, Belgium; ^2^BEAMS Department (Bio Electro and Mechanical Systems), Université Libre de Bruxelles, Brussels, Belgium; ^3^Cardiac Surgery Department, Universitair Ziekenhuis Brussel - Vrije Universiteit Brussel, Brussels, Belgium

**Keywords:** arrhythmias treatment, 3D printing, image processing, segmentation, Brugada syndrome

## Abstract

**Background:**

Brugada syndrome (BrS) is a disease associated with ventricular arrhythmias and sudden cardiac death. Epicardial ablation has demonstrated high therapeutic efficacy in preventing ventricular arrhythmias. The purpose of this research is to define a workflow to create a patient-specific 3D-printed tool to be used as a surgical guide for epicardial ablation in BrS.

**Methods:**

Due to their mechanical properties and biocompatibility, the MED625FLX and TPU95A were used for cardiac 3D surgical guide printing. ECG imaging was used to define the target region on the right ventricular outflow tract (RVOT). CT scan imaging was used to design the model based on patient anatomy. A 3D patient-specific heart phantom was also printed for fitting test. Sterilization test was finally performed.

**Results:**

3D printed surgical models with both TPU95A and MED625FLX models were in agreement with pre-specified imputed measurements. The phantom test showed retention of shape and correct fitting of the surgical tool to the reproduced phantom anatomy, as expected, for both materials. The surgical guide adapted to both the RVOT and the left anterior descending artery. Two of the 3D models produced in MED265FLX showed damage due to the sterilization process.

**Conclusions:**

A 3D printed patient-specific surgical guide for epicardial substrate ablation in BrS is feasible if a specific workflow is followed. The design of the 3D surgical guide ensures proper fitting on the heart phantom with good stability. Further investigations for clinical use are eagerly awaited.

## Introduction

Brugada syndrome (BrS) is a disease associated with ventricular arrhythmias and sudden cardiac death. A mutation in the SCN5A gene, which codes for sodium channels, can be found in 20–25% of BrS patients (1, 2). The prevalence of BrS is ≈5–20 per 10,000 subjects worldwide and accounts for ≈20% of sudden death in patients with apparently normal hearts (3). Several therapeutic strategies have been developed over the years, including the following: drug treatment, implantable cardioverter-defibrillator (ICD), and catheter ablation. Among these solutions, catheter ablation has demonstrated high therapeutic efficacy in preventing ventricular arrhythmias with complete elimination of the BrS ECG phenotype (4) and the abnormal epicardial substrate in the right ventricular outflow tract (RVOT) (5–7). ECG imaging (ECGI) is a non-invasive mapping system used to assess the epicardial substrate in BrS (8); this tool is based on high-density electrocardiography that reconstructs epicardial potentials from ECG signals (8). It can give information on the anatomy and extent of the abnormal substrate in BrS that should be targeted on the epicardium of RVOT. However, the clinical images used to detect the abnormal area cannot be directly applied as a guide during epicardial ablation (open chest, mini thoracotomy or hybrid thoracoscopic) (9).

The purpose of this research is to define a workflow to create a patient-specific 3D-printed tool to be used as a surgical guide for epicardial ablation in BrS. In particular, in this paper, the printing process is described for a case of Brugada syndrome (index case). Furthermore, a literature review is provided.

## Methods

### Data acquisition and images processing

The first step to create a 3D guide for BrS epicardial ablation was to acquire an ECGI map. CardioInsight (Medtronic Inc, Minneapolis, MN) non-invasive 3D mapping system technology was used for this aim. In BrS, the results of invasive mapping studies have been shown to be consistent with those obtained with ECGI (5, 10–12).

ECGI is able to determine the electrical activity of the heart non-invasively, by reconstructing epicardial potentials from signals obtained from the trunk. In particular this is performed solving the inverse problem of electrocardiography (13) by subsequent processing of signals using mathematical reconstruction algorithms. An ECGI vest consisting of 252 electrodes is attached to the patient's torso and connected to a CardioInsight system that records the signals of 252 unipolar electrocardiograms. The anatomy of the heart and the position of each electrode on the trunk is reconstructed from the patient-specific CT scan. The chamber geometry is then reconstructed to obtain a three-dimensional model. This model is used to project unipolar signals represented by virtual nodes onto the epicardial surface (14). Additional signal processing allows the generation of different types of maps, such as activation maps, voltage maps, iso-potential maps, and phase maps. The UZBCIT software (Medtronic Inc, Minneapolis, MN) enables simultaneous visualization of the ventricular volume and mapping of the electrical activity of the heart.

For the purpose of this study, mapping was performed with the CardioInsight Non-invasive 3D Mapping System (Medtronic Inc, Minneapolis, MN) in one BrS patient (index patient). Unipolar electrocardiograms obtained from the 252 electrodes Vest were projected into 3D geometry. Activation time (AT), repolarization time (RT), and activation-repolarization interval (ARI) maps were automatically obtained using CardioInsight technology. The activation time for each unipolar electrogram was defined as the time elapsed between the onset of depolarization (QRS) and the maximum negative slope (maximum negative dV/dt) of the electrogram. Activation delay boundaries were delineated through a post-analytical process using a next-generation software (UZBCIT) developed by Medtronic Inc. (Minneapolis, MN), which can provide a quantitative measure of the pathological RVOT area, based on activation delay.

**Figure 1:**
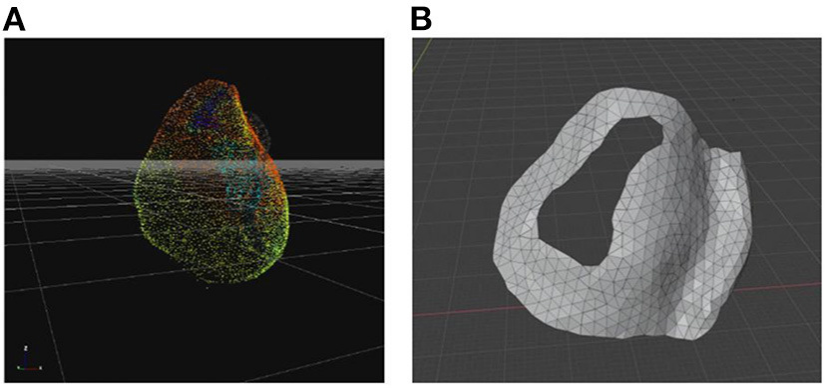
3D models for surgical guide printing. **(A)** Photogrammetric reconstruction view in Zephir software scatter point; **(B)** Tool model in Blender software.

As a second step the images derived from ECGI (UZBCIT software) were processed with photogrammetry using the free version of ZephirLite (3DFlow©). Zephir works semi-automatically. After the photos are loaded, the masks obtained with Masquerade are loaded and the parameters for the reconstruction are set. In this case, the reconstruction was obtained through the high-resolution option for a general object. First, the software creates a cloud of scattered points (Figure 1), which are obtained automatically by placing a point in three or more images and then generating a dense cloud, on which a densification filter has been applied to obtain a better visualization of the cloud itself. From this cloud, the software creates a network to which a topology optimization filter has been applied. Finally, the textured mesh is exported to the saved.obj. This image processing takes about 2 h.

### Surgical prototype design

Blender 3.0 (Blender Foundation, Amsterdam, The Netherlands) was used to design the model for the index patient (Figure 1). The first step was to get a mesh of RVOT that is based on patient anatomy; to achieve this, the reference measurements were derived from the photogrammetry-based reconstruction exported from ZephirLite (3DFlow©) (15). Blender software can scale up or down a 3D mesh evenly throughout its volume. The 3D object can be enlarged or reduced, the geometry can be refined and the thickness can be adjusted through the 3D extrusion process. Once the correct dimensions were obtained, the mesh was manipulated using the Modeling > Knife function to remove excess parts and shape the opening (surgical hole) for the arrhythmogenic region of interest. As next step, the function Modeling > Extrude Along Normal was used to create a tool thickness of 1 mm. The region of interest was identified by a previous ECGI map examination, together with an experienced cardiologist on the basis of the latest activated area.

Once the surgical guidance was defined, smoothing functions were used to create an appropriate design of the 3D model. To ensure stable placement of the surgical guide on the heart, the left anterior descending coronary artery (Figure 1) was chosen as a reference anatomical landmark. This was considered a point where a guide could be placed on the organ to ensure its correct position. After the mesh manipulation was completed, the mesh was prepared for printing using two steps. The first step was the Layout> 3D-print> checks function, which allows detecting possible errors in the mesh, and then using the Layout> 3D-print>Clean-up> makemainfold function, which allows to clean up issues like holes, non-major folding, and inverted normals. At this point, the model was extracted using Layout > 3D-print > export to.STL format (Figure 1).

### 3D prototype printing

**Figure 2:**
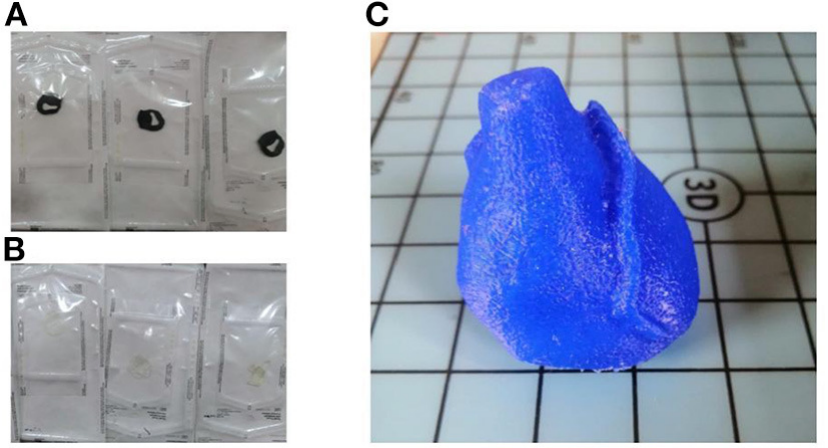
3D surgical guides and 3D heart phantom. **(A)** Printed and cleaned surgical tool in TPU95A; **(B)** Printed and cleaned surgical tool in MED625FLEX; **(C)** Printed and cleaned 3D model of the phantom in TPU95A.

The 3D surgical model for the index patient was printed using two different techniques with two different materials. A pre-specified thickness of 1 mm was chosen. The FDM technique was used to create the model using Anycubic's Mega Zero printer. The model was uploaded to Cura (Ultimaker) software, which is necessary to prepare the model for printing. Subsequently, the parameters for printing were set: the extruder speed was set to 20 mm/s (16) for higher accuracy, the extrusion temperature was set to 225°C, and the filling percentages were set to 100%. The sample was printed using TPU95A material (Figure 2).

The other technique used was the polyjet; it is a hybrid of selective curing and droplet deposition. After spraying each layer, the tray is moved down one layer in thickness, then the process is repeated with the next layer (17). The most important component is the printhead: it applies a liquid compound of reactive monomers and oligomers that polymerize in response to ultraviolet light. The 3D printer used was a Stratasys Object260 Connex1 (Stratasys Inc., Eden Prairie, MN, USA). The material chosen for this research was a flexible, transparent, biocompatible material from Stratasys: MED625FLX TM (Stratasys Inc., Eden Prairie, MN, USA). The model was printed in monomaterial mode and the printer was set to glossy printing to obtain a thicker layer (Figure 2). The thickness of each layer measured 16 nanometers; therefore, this process can be classified among the most accurate processes, and the device can be considered one of the fastest processes for rapid prototyping. The small thickness of the layers ensured the production of a model with a very smooth surface and small details. The material, substrate and print mode were defined before the print was started. Once all the settings were selected, the software calculated the print time based on the position of the object on the tray. After the printing process was completed, the surgical guide was inserted into the SUP706 holder (FullCure705), a gel-like carrier material that was then carefully removed. A high-pressure water jet was used to remove the carrier material (Objet WaterJet cleaning unit, Stratasys Inc., Eden Prairie, MN, USA). The cleaning time varies for each printed model depending on its design.

Sterilization resistance tests were performed at the UZ Brussel Hospital to assess if the samples could be damaged during sterilization. The sterilization procedure is a mandatory step for the practical use of a surgical guide. To evaluate the response of the 3D printed object to sterilization, four samples with different thicknesses were selected for each material: 1, 1.2, 1.5, and 2.2 mm, respectively. Sterilization by vapor peroxide (VHP) with a STERRAD NX sterilizer (Johnson & Johnson, Irvine, CA) with a standard setting of 43°C for 43 min was chosen.

To evaluate the impact of the sterilization process on the 3D printed models, the samples were classified in three categories, according to the degree of damage to the prototype during sterilization (Table 1). The three classes have been developed taking into account the requirements for the final result.

**Table 1:** Categories of damage to the 3D surgical model during sterilization.

**Category**	**0**	**1**	**2**
Damage	Upon visual inspection, no material breakage is present. The geometry is intact and macroscopically unmodified.	Upon visual inspection, material breakage is present. The geometry is intact and macroscopically unmodified.	On visual inspection, there is evident damage to the material. The geometry is completely altered as it is macroscopically broken into several pieces.

Three different categories are described for the physical changes undergone by the tool during the sterilization process.

A 3D model of the patient's heart anatomy (specific for the index patient) was printed at full scale as a phantom to test the placement of the surgical guide (Figure 2). The printing process chosen to manufacture the phantom was fused deposition (FDM) using a Mega zero 3D printer.

Thermoplastic polyurethane (TPU95A) was used. The phantom included the RVOT and the left anterior descending artery, thus being suitable to test surgical guide placement.

## Results

After the printing process was completed, the support material was removed; removing the support did not cause any damage to the models. The models showed no signs of mechanical stress and no printing errors. Printing and model cleaning were completed in a 5 h cycle.

3D printed surgical models both with TPU95A and MED625FLX measured 1 mm in thickness, as expected.

The TPU95A model weighed 6 grams and measured 59.79 mm in width, 33.57 mm in length and 45.31 mm in height. The model had a volume space of 6.09 mm^3^. FDM printing was done in high mode with a total TPU95A consumption of 36 g (Figure 2).

The MED625FLX model weighted 6.5 grams and measured 59.79 mm in width, 33.57 mm in length and 45.31 mm in height. The model had a volume space of 6.09 mm^3^. Polyjet printing was done in high mode with a total consumption of 10.507 ml (Figure 2).

The region of interest (surgical hole) of the printed model measured 27 mm in height and 15.1 mm in width; measurements were consistent with pre-specified targets imputed in Blender software.

**Figure 3:**
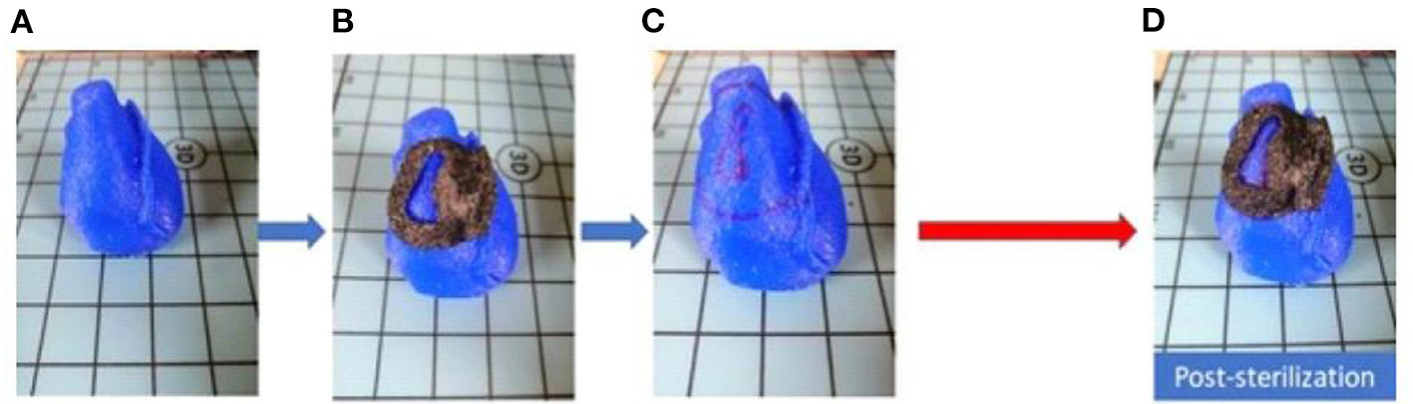
Fitting test pre and post sterilization. **(A)** Phantom in full scale; **(B)** pre-sterilization fitting test; **(C)** Phantom with applied 3D model edges marked; **(D)** post-sterilization fitting test.

The phantom fit test showed shape retention and proper fit of the surgical tool to the reproduced phantom anatomy, as expected, for both materials (Figure 3). The surgical guide fitted both the RVOT and left anterior descending artery. The addition of the anatomical landmark allowed high stability of the 3D surgical guide positioning.

**Figure 4:**
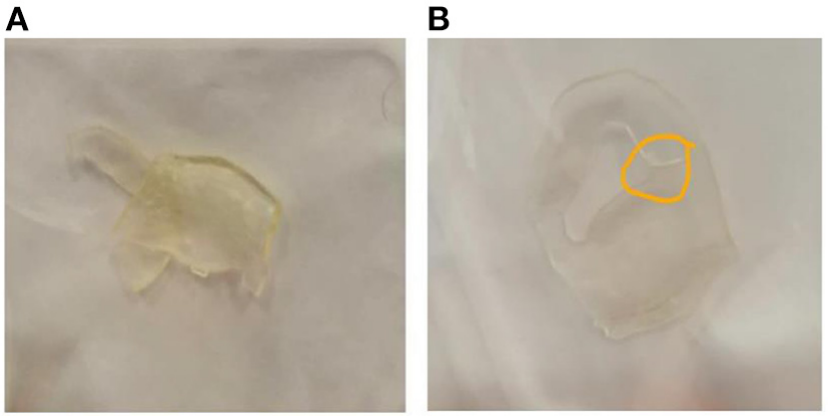
Damaged samples during the sterilization process. **(A)** prototype in MED625FLX 2.2 mm damaged during sterilization; **(B)** prototype in MED625FLX 1 mm damaged during sterilization.

The assessment of 3D surgical samples after sterilization is summarized in Table 2. In particular, TPU95A-based models did not show any damage. Models in MED625FLX (1 and 2.2 mm) were damaged during the sterilization procedure (Figure 4). The samples printed with both materials that were unaffected by the sterilization step retained their pre-sterilization dimensions, according to the 3D design.

**Table 2:** Results of the sterilization process.

**Thickness**	**Post-sterilization visual results for printed TPU95A models**	**Post-sterilization visual results for printed MED625FLX models**
1 mm	0	1
1.2 mm	0	0
1.5 mm	0	0
2.2 mm	0	2

The table summarizes the physical changes of the samples based on the three defined categories.

## Discussion

This study is the first to demonstrate the feasibility of a patient-specific surgical guide for epicardial substrate ablation in BrS. The workflow to print a 3D surgical guide for patients suffering from BrS and candidates to substrate ablation is described for an index case.

Surgical guidance aims at protecting the healthy part of the heart tissue, thus ensuring targeting directly the specific pathogenic area in the RVOT of BrS patients.

An effective 3D surgical guide for BrS should respect the following criteria: (1) patient specific anatomy and (2) targeting of the patient specific abnormal region in the RVOT. This can be performed using CT scan imaging imported in the UZBCIT software. ECGI is of utmost importance to localize the region of interest. Finally, Zephyr Lite software (3DFlow©) has been used to create the 3D volume. Using this software, it was possible to create a 3D mesh starting from jpg images. Two printing techniques (Polyjet or FDM) were suitable for its use.

The use of Polyjet printers offers shorter production times and better surface treatment of printed objects than the FDM technique. On the other hand, FDM technology is more affordable than Polyjet, and the selection of materials with Polyjet technology, despite being tested and approved for medical use, is severely limited compared to the variety found in FDM manufacturing.

MED625FLX is a flexible, transparent and biocompatible material. Two of the 3D models produced in MED265FLX showed damage due to the sterilization process, but given the location of the damaged parts, it is possible that the damage was due to the geometry of the prototype rather than the thickness or the material.

Flexible TPU95A is available on the market at a relatively affordable price, the printing technique is readily available and its mechanical and physical properties make it suitable for the intended type of medical use.

### Literature review

Previous studies demonstrated the feasibility of a surgical guide for coronary artery bypass graft, to visualize the region of interest for bypass placement during the intervention (18). In particular this 3D model can be used to visually target the coronary artery stenosis for bypass placement overcoming the epicardial fat which can prevent an optimal view; a bypass model can be used both as a guide and as a surgical educational tool.

The material for printing is a crucial step when coming to bioprinting and biocompatibility.

Xiao et al. showed that TPU95A has higher tensile strength and elongation when printed at temperatures between 215 and 230°C than at lower temperatures. They also demonstrated that if the extrusion temperature is in the above range, there is an increase in thermal coupling between the edges and the interior of the sample, which dramatically reduces the possibility of sample breakage (17).

For the hereby presented 3D model MED625FLX and TPU95A were chosen because of previous literature evaluating the effect of radiofrequency energy sources. In particular both materials have been recently tested by our group with radiofrequency catheter ablation (19). For surgical guides design, the 2.5 mm MED625FLX could be used, with bipolar radiofrequency catheter, ensuring good geometrical, mechanical and thermal properties. None of the two biomaterials tested are suitable for unipolar radiofrequency ablation.

Samples printed with both materials that remained intact after sterilization and showed dimensional consistency. The design of the 3D surgical guide ensured proper fitting on the heart phantom with good stability. Although the MED625FLX was specially designed by the manufacturer for medical use, its elongation rupture is not extremely high, so it might be better suitable for mini thoracotomy. TPU95A material is more flexible and has a greater elongation when sliding, thus it might be suitable for use during thoracoscopic catheter ablation.

### Future perspective

The hereby described workflow has been applied for one case (index) patient with BrS. Assessing the reproducibility of the described 3D model in a large cohort is eagerly awaited. Future research is needed to test mechanical properties and further assess biocompatibility. In particular, the response of the material to radiofrequency ablation has been evaluated but further evaluation for cryoablation is needed.

## Conclusions

A 3D printed patient-specific surgical guide for epicardial substrate ablation in BrS is feasible if a specific workflow is followed. The design of the 3D surgical guide ensures proper fitting on the heart phantom with good stability. Further investigations for clinical use are eagerly awaited.

## Data availability statement

The original contributions presented in the study are included in the article/supplementary material, further inquiries can be directed to the corresponding author.

## Ethics statement

The studies involving human participants were reviewed and approved by Commissie Medische Ethiek UZ Brussel. The patients/participants provided their written informed consent to participate in this study.

## Author contributions

GT, LP, BI, and CA: conception and design of the work. LP, CM, GT, and RR: substantial contributions to the acquisition of data for the work. LP, CM, EB, IC, and MC: substantial contributions to the analysis of data for the work. RR, ML, AG, GC, BI, and CA: substantial contributions to the interpretation of data for the work. GT and LP: drafting the work. RR, ML, AG, GC, BI, and CA: revising the draft of the work critically for important intellectual content. GT, LP, CM, EB, IC, MC, RR, ML, AG, GC, BI, and CA: final approval of the version to be published. GT, LP, CM, EB, IC, MC, RR, ML, AG, GC, BI, and CA: agreement to be accountable for all aspects of the work in ensuring that questions related to the accuracy or integrity of any part of the work are appropriately investigated and resolved. All authors contributed to the article and approved the submitted version.

## Conflict of interest

Author ML is consultant for Atricure. Author GC received compensation for teaching purposes and proctoring from Medtronic, Abbott, Biotronik, Boston Scientific, Acutus Medical. Author CA receives research grants on behalf of the center from Biotronik, Medtronic, Abbott, LivaNova, Boston Scientific, AtriCure, Philips, and Acutus; Author CA received compensation for teaching purposes and proctoring from Medtronic, Abbott, Biotronik, Livanova, Boston Scientific, Atricure, Acutus Medical Daiichi Sankyo. The remaining authors declare that the research was conducted in the absence of any commercial or financial relationships that could be construed as a potential conflict of interest.

## Publisher's note

All claims expressed in this article are solely those of the authors and do not necessarily represent those of their affiliated organizations, or those of the publisher, the editors and the reviewers. Any product that may be evaluated in this article, or claim that may be made by its manufacturer, is not guaranteed or endorsed by the publisher.
